# Antigen Detection in Urine for Noninvasive Diagnosis and Treatment Monitoring of Visceral Leishmaniasis in Human Immunodeficiency Virus Coinfected Patients: An Exploratory Analysis from Ethiopia

**DOI:** 10.4269/ajtmh.18-0042

**Published:** 2018-08-06

**Authors:** Florian Vogt, Bewketu Mengesha, Helen Asmamaw, Tigist Mekonnen, Helina Fikre, Yegnasew Takele, Emebet Adem, Rezika Mohammed, Koert Ritmeijer, Wim Adriaensen, Yayehirad Melsew, Johan van Griensven, Ermias Diro

**Affiliations:** 1 Department of Clinical Sciences, Institute of Tropical Medicine Antwerp, Antwerp, Belgium;; 2 Leishmaniasis Research and Treatment Center, University of Gondar, Gondar, Ethiopia;; 3 Gondar University Hospital, University of Gondar, Gondar, Ethiopia;; 4 Public Health Department, Médecins Sans Frontières, Amsterdam, The Netherlands;; 5 Department of Epidemiology and Biostatistics, University of Gondar, Gondar, Ethiopia

## Abstract

Diagnosis of visceral leishmaniasis (VL) and assessment of treatment response in human immunodeficiency virus (HIV)–coinfected patients still relies on invasive tissue aspiration. This hampers scale-up and decentralization of care in resource-limited settings. Noninvasive diagnostics are urgently needed. KATEX is a frequently used latex agglutination test for *Leishmania* antigen in urine that has never been evaluated in HIV-coinfected individuals from *Leishmania donovani*–endemic areas. This was an exploratory sub-study embedded within the screening phase of a trial in highly endemic northwestern Ethiopia. All patients were HIV-positive and aspirate-confirmed VL cases. We assessed diagnostic accuracy of KATEX for VL diagnosis and as test of cure at end of treatment, using tissue aspirate parasite load as reference methods. We also described the evolution of weekly antigen levels during treatment. Most of the 87 included patients were male (84, 97%), young (median age 31 years), and had poor immune status (median cluster of differentiation type 4 count 56 cells/μL). KATEX had moderate sensitivity (84%) for VL diagnosis. KATEX had moderate sensitivity (82%) and a moderate negative predictive value (87%) but only low specificity (49%) and a low positive predictive value (40%) for the assessment of treatment outcomes. Weekly antigen levels showed characteristic patterns during treatment of patients with different initial parasite loads and treatment outcomes. Antigen detection in urine using KATEX can contribute to improved VL diagnosis in HIV-coinfected patients but has limited use for monitoring of treatment response. Better noninvasive diagnostics are needed to reduce reliance on invasive methods and thus to expand and improve clinical care for VL in resource-limited settings.

## INTRODUCTION

Coinfection of human immunodeficiency virus (HIV) and visceral leishmaniasis (VL) is an emerging problem worldwide, with high or increasing incidence reported from Ethiopia, Brazil, and India.^1,2^ The highest VL/HIV coinfection prevalence worldwide can be found in northwestern Ethiopia, with up to 20% of VL patients being HIV coinfected.^1,3–5^ Visceral leishmaniasis is universally lethal if left untreated. A rapid deterioration is often observed in HIV-coinfected patients.^3,6^

Visceral leishmaniasis diagnosis is particularly problematic in this group because antibody-based serology markers perform poorly because of suppressed antibody production.^3,7–9^ Therefore, invasive parasitological confirmation from spleen or bone marrow aspiration is still widely practiced in HIV patients, particularly in resource-limited settings.^1,10^ Given the high rate of treatment failure and the unreliability of clinical assessment for treatment response in HIV-coinfected patients,^11^ the same invasive procedure is typically repeated once or several times to confirm parasitological cure.^1,12^ This practice is not only painful but also requires substantial level of training and is prone to potentially fatal complications such as hemorrhage, thereby limiting it to referral hospitals.^1,13,14^ Misdiagnoses are common where capacity for invasive diagnostic does not exist.

To overcome these shortcomings, noninvasive antigen tests have been developed for VL diagnosis.^13–15^ KATEX (Kalon Biological Ltd., Guilford, United Kingdom)^16^ is a frequently used urine-based antigen test kit in resource-limited settings.^17,18^ Evaluations in immunocompetent patients show good specificity but only moderate sensitivity.^7^ There is also evidence that antigen clearance in urine correlates with tissue *Leishmania* parasite load and treatment response.^19–21^ Because of impaired immune response combined with abundant antigenemia, detecting antigens rather than the serological response against the parasite could yield a better sensitivity in HIV-coinfected patients compared with HIV-negative individuals.^11^ In Europe, where *Leishmania infantum* is prevalent, there are indications from a couple of small studies conducted over 10 years ago that KATEX sensitivity might indeed be higher in HIV coinfected than in immunocompetent patients.^22,23^ However, none of this has been studied in HIV-coinfected patients from *Leishmania donovani*–endemic settings.

Urine antigen tests have the potential to reduce the reliance on invasive procedures for VL diagnosis and treatment monitoring, in particular for HIV-coinfected patients. This would allow scale-up and decentralization of care in high-burden resource-limited settings such as Ethiopia.^1^ We aimed to explore the performance of KATEX urine antigen testing for VL diagnosis and monitoring of treatment response in HIV-coinfected patients in northwestern Ethiopia. Specifically, our objectives were to 1) assess sensitivity of KATEX for VL diagnosis, 2) describe the evolution of antigen levels during treatment, and 3) assess diagnostic accuracy of KATEX as test of cure (TOC), with microscopic examination of tissue aspirates as reference method.

## MATERIAL AND METHODS

### Study design and population.

This was a diagnostic sub-study nested within the screening phase of a clinical trial that assessed the safety and efficacy of pentamidine to prevent VL relapse in HIV-coinfected patients in Ethiopia (clinicaltrials.gov identifier NCT01360762).^24^ In that trial, patients were enrolled and received the intervention (pentamidine as secondary prophylaxis) only after successful completion of their VL treatment. The VL treatment phase was hence part of the trial screening period. We used samples collected during this screening phase, that is, before participants received the trial intervention.

All patients included in this analysis were aged ≥ 18 years, HIV positive, had a parasitologically confirmed VL episode (tissue aspirates obtained for all patients), and started VL treatment. Recruitment lasted from November 2011 to July 2013. Patients originated from high VL burden areas in northwestern Ethiopia and were treated for VL as inpatients.

### Setting.

Trial recruitment sites were the Leishmaniasis Research and Treatment Center of the University of Gondar, and the Abdurafi Health Center, Amhara region, Ethiopia. Both sites function as important VL treatment centers in the region. The Leishmaniasis Research and Treatment Center is supported by the Drugs for Neglected Diseases initiative, whereas the Abdurafi Health Center is supported by Médecins sans Frontières (MSF).

### Procedures.

A VL-focused clinical examination and venous blood sample collection was carried out at the beginning and end of the VL treatment. Urine was collected at treatment start, weekly during the treatment, and at the end of VL treatment. Visceral leishmaniasis treatment followed national and MSF guidelines.^25,26^ The treatment duration was 30 days for most patients and 17 days for patients on a sodium stibugluconate/paromomycin combination regimen. Tissue aspirates from spleen, bone marrow, or lymph nodes were obtained from all patients at treatment start to confirm VL diagnosis parasitologically and at the treatment end as TOC.

Aspirate preparation and examination followed national and World Health Organization guidelines.^25,27^ In short, slide smears were prepared directly after collection, air-dried, fixed with methanol, and Giemsa-stained. Parasite load was determined through microscopic examination under 100× oil immersion as amastigote density ranging from 0 (negative) to +6 (> 100 parasites per field). For KATEX testing, 5 mL of urine was collected in sterile containers, aliquoted, and stored at −20°C. The assay is based on the detection of a low molecular weight antigen (5–20 kDa) associated with the nitrocellulose membrane through agglutination with polystyrene latex particles coated with polyclonal anti-*L. donovani* antigen antibodies.^28,29^ Testing followed manufacturer’s instructions^16^: In short, 50 μL of latex solution containing antibody-coated beads was added to 50 μL of heat-inactivated urine. Agglutination levels were assessed visually after 2 minutes and graded as follows: weakly positive (+1) if agglutination could just be discerned when compared with the negative control, moderately positive (+2) if agglutinated particles could clearly be seen against a background of granular latex, and highly positive (+3) if the latex had agglutinated and collected around the edge of the reaction zone. No visible agglutination compared with negative control was considered as negative. Findings were verified independently by two experienced laboratory technicians.

### Data analysis.

Percentages and medians of sociodemographic, HIV-related, and VL treatment–related variables were collated for all included patients. Continuous variables were categorized according to commonly used cutoffs. For the diagnostic accuracy analyses, both KATEX and aspirate results were categorized as binary (positive/negative), and 95% confidence intervals (95% CI) were calculated.

For VL diagnosis, the correlation between aspirate and KATEX results at treatment start was plotted and summarized using Spearman’s Rho correlation coefficient. Sensitivity of KATEX for VL diagnosis was calculated by comparing KATEX results at treatment start (index test) with aspirate results at treatment start (reference test) across selected sociodemographic and clinical characteristics. Because all patients had parasitologically confirmed VL, specificity could not be calculated within this group. We used healthy endemic controls to calculate specificity for laboratory quality control purposes.

For antigen evolution during treatment, aspirate results at the beginning and end of treatment were plotted against KATEX results at treatment start. The distribution of treatment outcomes by KATEX results at treatment start was compared and summarized using Fisher’s exact test. Weekly antigen levels during treatment were plotted graphically by aspirate results at treatment start and by treatment outcome.

For TOC, the correlation between aspirate and KATEX results at treatment end was plotted and summarized using Spearman’s Rho correlation coefficient. Sensitivity, specificity, positive predictive values (PPVs), and negative predictive values (NPVs) of KATEX as TOC were calculated by comparing KATEX results at treatment end (index test) with aspirate results at treatment end (reference test) across sociodemographic and clinical characteristics.

We used the software package STATA version 14.2 (StataCorp., College Station, TX) for all analyses.

### Ethics.

This study was conducted as a sub-study based on samples and data generated during the conduct of the clinical trial “Prophylaxis of Visceral Leishmaniasis Relapses in HIV Co-infected Patients with Pentamidine: a Cohort Study” (clinicaltrials.gov identifier NCT01360762) as outlined in the original study protocol. The trial was approved by all concerned ethics and administrative committees, namely, the Ethiopian Food, Medicine, Health Care Administration and Control Authority; the Ethiopian National Research Ethics Review Committee; the Institutional Review Board of the University of Gondar; the Ethics Review Board of MSF; the Ethics Committee of Antwerp University Hospital; and the Institutional Review Board of the Institute of Tropical Medicine, Antwerp. Written informed consent was obtained from all participants. Visceral leishmaniasis treatment was provided free of charge.

## RESULTS

A total of 87 patients with parasitologically confirmed VL were included in the analysis (see Supplementary Material 1 for patient selection by study objective). Most patients were male (84/87, 97%), young adults (median age 31 years), with recent HIV diagnosis (median duration 5.4 months), and with a low cluster of differentiation type 4 counts (median 56 cells/μL). Primary VL accounted for 59% of cases. At treatment start, 42 (58%) patients had highly positive (+3) KATEX results, and 33 (38%) had very high (+6) aspirate parasite loads. At treatment end, 52 (70%) patients achieved cure and 23 (38%) were KATEX negative (Table 1).

**Table 1:** Patient and treatment characteristics

Patient characteristics	*N* = 87	%*
Sex		
Female	3	3.4
Male	84	96.6
Age		
Median, IQR (years)	31	8
≤ 30 years	42	48.3
> 30 years	45	51.7
Time since HIV diagnosis (*n* = 81)		
Median, IQR (months)	5.4	13.8
< 1 months	26	32.1
1–12 months	31	38.3
> 12 months	24	29.6
CD4 cell count (*n* = 76)		
Median, IQR (cells/μL)	56	70
≤ 50 cells/μL	35	46.1
> 50 to ≤ 150 cells/μL	30	39.5
> 150 cells/μL	11	14.5
ART status (*n* = 75)		
On ART	47	62.7
Not on ART	28	37.3
Time since ART initiation (*n* = 47)		
Median, IQR (months)	6.1	12.8
< 3 months	11	23.4
3–12 months	22	46.8
> 12 months	14	29.8
Number of previous VL episodes		
0	51	58.6
1	25	28.7
2	9	10.3
3	2	2.3
KATEX result (*n* = 73)		
0	12	16.4
1	10	13.7
2	9	12.3
3	42	57.5
Aspirate result		
1	8	9.2
2	12	13.8
3	10	11.5
4	13	14.9
5	11	12.6
6	33	37.9
KATEX result, at treatment end (*n* = 61)		
0	23	37.7
1	13	21.3
2	6	9.8
3	19	31.1
Treatment outcome, at treatment end (*n* = 74)		
Cure	52	70.3
Failure; aspirate result, at treatment end	22	29.7
1	5	22.7
2	3	13.6
3	4	18.2
4	6	27.3
5	1	4.5
6	3	13.6

ART = anti-retroviral therapy; CD4 = cluster of differentiation type 4; HIV = human immunodeficiency virus; IQR = interquartile range; *N* = total; *n* = subtotal excluding records with missing values; VL = visceral leishmaniasis. All variables refer to the time of treatment start unless stated otherwise.

* Percentage of column total.

**Figure 1.**
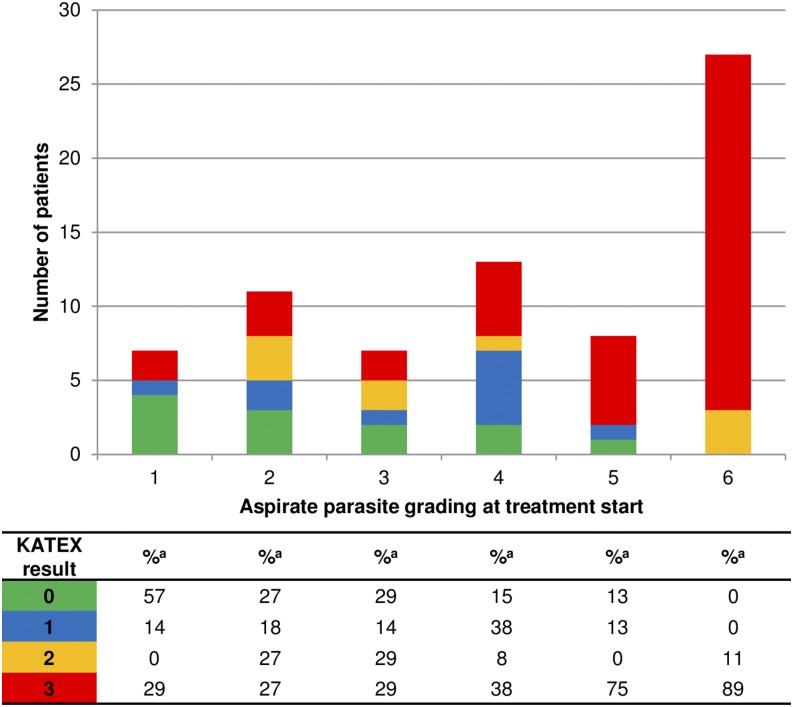
Aspirate and KATEX results at treatment start. Fourteen patients with missing KATEX result at treatment start excluded. ^a^ Percentage of column total. This figure appears in color at www.ajtmh.org.

**Figure 2.**
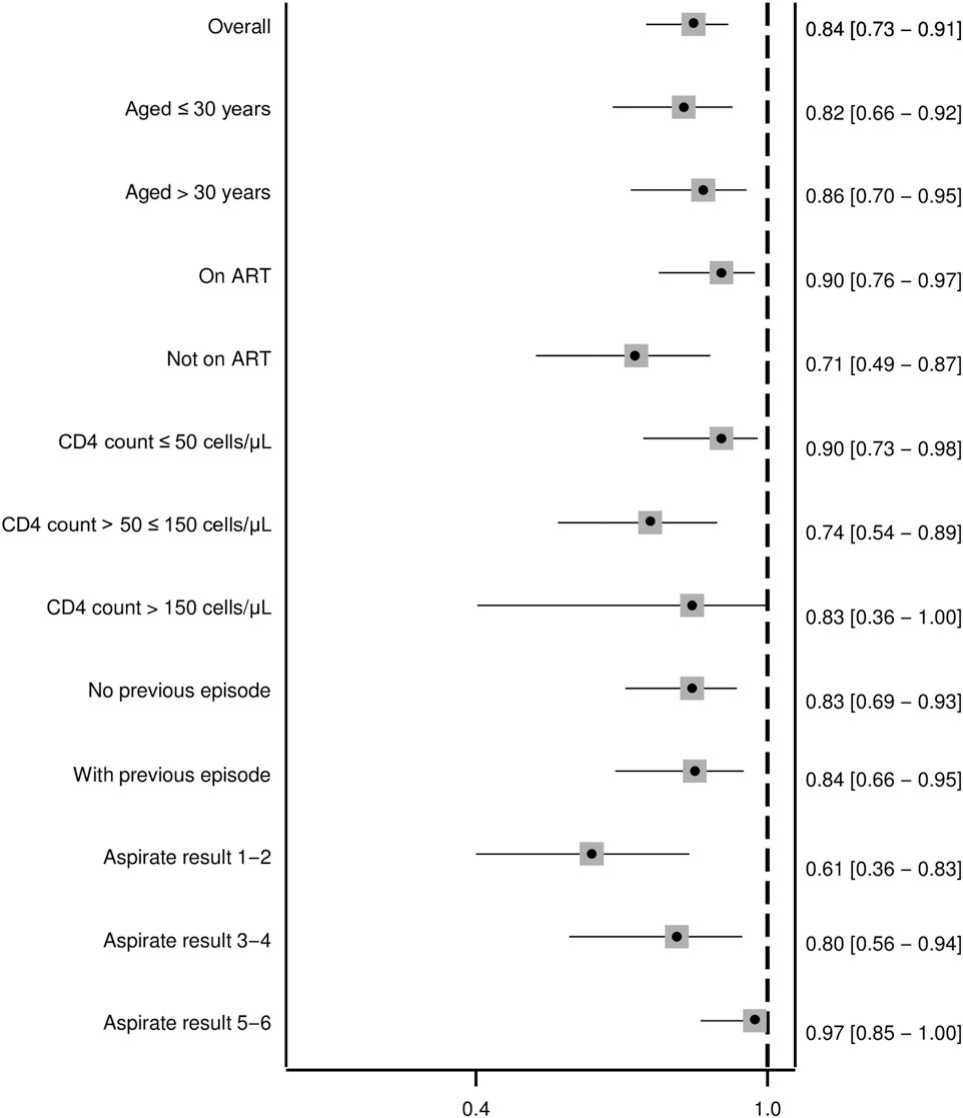
Sensitivity of KATEX at treatment start for visceral leishmaniasis diagnosis. Fourteen patients with missing KATEX result at treatment start excluded (see Supplementary Material 1). All variables refer to the time of treatment start. 95% confidence intervals in brackets.

There was a notable correlation between KATEX and aspirate results at treatment start which was mostly due to clustering of highly positive (+3) KATEX results in aspirates with highest (+6) parasite loads (Figure 1). This was confirmed by a Spearman’s Rho correlation coefficient of 0.569 (*P* value < 0.001). A substantial variation of KATEX results in patients with low or medium aspirate parasite loads was observed (Figure 1). Overall sensitivity of KATEX for VL diagnosis was moderate (84%, 95% CI: 73–91). Across stratified covariates, only aspirate parasite load was positively associated with sensitivity, with patients having a higher parasite load being more likely to be diagnosed by KATEX (Figure 2).

**Figure 3.**
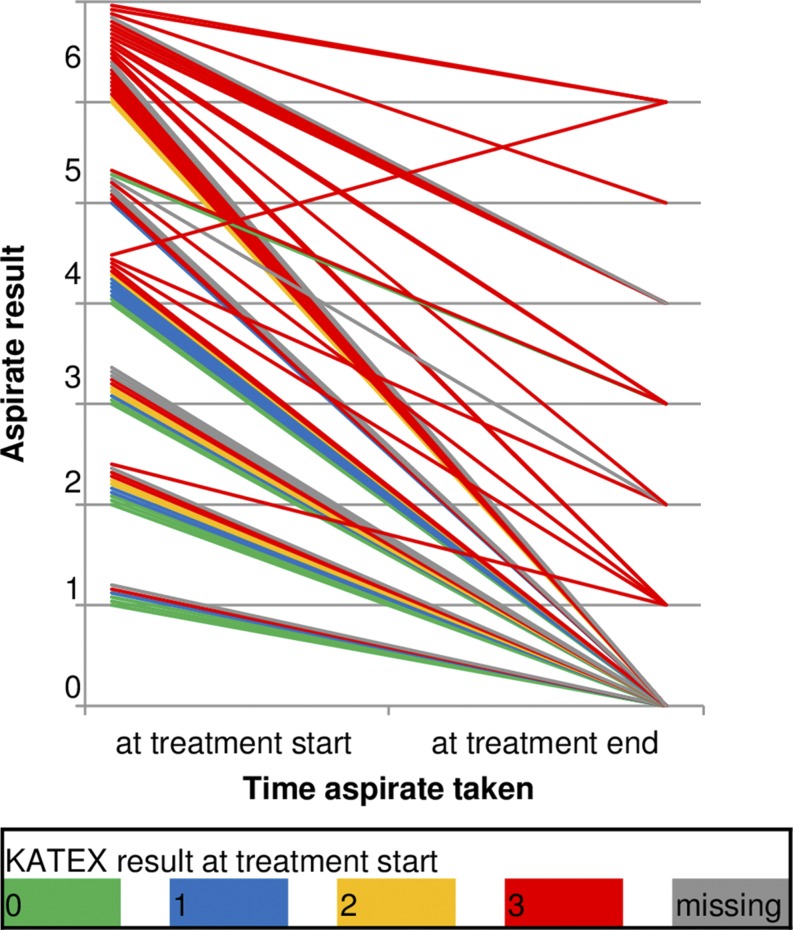
Aspirate results at start and end of treatment by KATEX result at the treatment start. Thirteen patients with missing aspirate result at treatment end excluded (see Supplementary Material 1). This figure appears in color at www.ajtmh.org.

**Figure 4.**
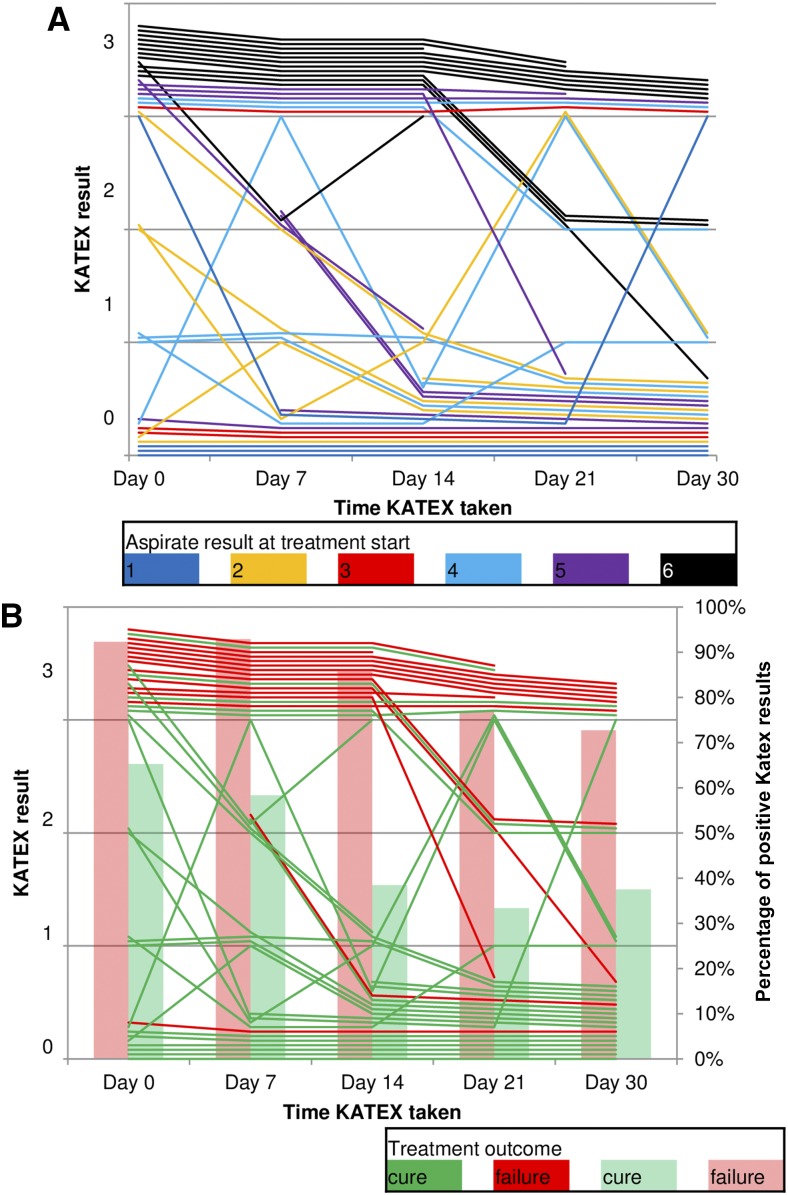
Evolution of KATEX results during treatment. (**A**) By aspirate result at treatment start and (**B**) by the treatment outcome. Thirteen patients with missing aspirate result at treatment end, 28 patients with less than three consecutive KATEX results during treatment, and six patients with short treatment duration excluded (see Supplementary Material 1). This figure appears in color at www.ajtmh.org.

Parasite load reduced during treatment in nearly all patients irrespective of initial KATEX result (Figure 3). Except one, all patients with subsequent treatment failure had already high (+3) KATEX results at treatment start (Table 2). Fisher’s exact test for this association was highly significant (*P* value < 0.001). Weekly monitoring of KATEX results showed relatively stable trends throughout treatment in patients with both very high (+6) and very low (+1) parasite loads at treatment start, but substantial variation was observed for medium ranges (Figure 4A). Most cured patients were KATEX negative after 2 weeks of treatment, although fluctuations during the second half of the treatment were not uncommon. The proportion of KATEX positivity during treatment declined in patients with subsequent treatment failure and cure alike but remained on different levels (from 92% to 73% among treatment failures, and from 65% to 38% among cured patients) (Figure 4B).

**Table 2:** KATEX results at the treatment beginning and treatment outcomes

	Cure		Treatment failure	
	*n*	%*	*n*	%*
KATEX result at the treatment beginning				
0	10	19.2	1	4.5
1	10	19.2	0	0.0
2	7	13.5	0	0.0
3	16	30.8	19	86.4
Missing	9	17.3	2	9.1

Thirteen patients with missing aspirate result at treatment end excluded (see Supplementary Material 1).

* Percentage of column total.

**Figure 5.**
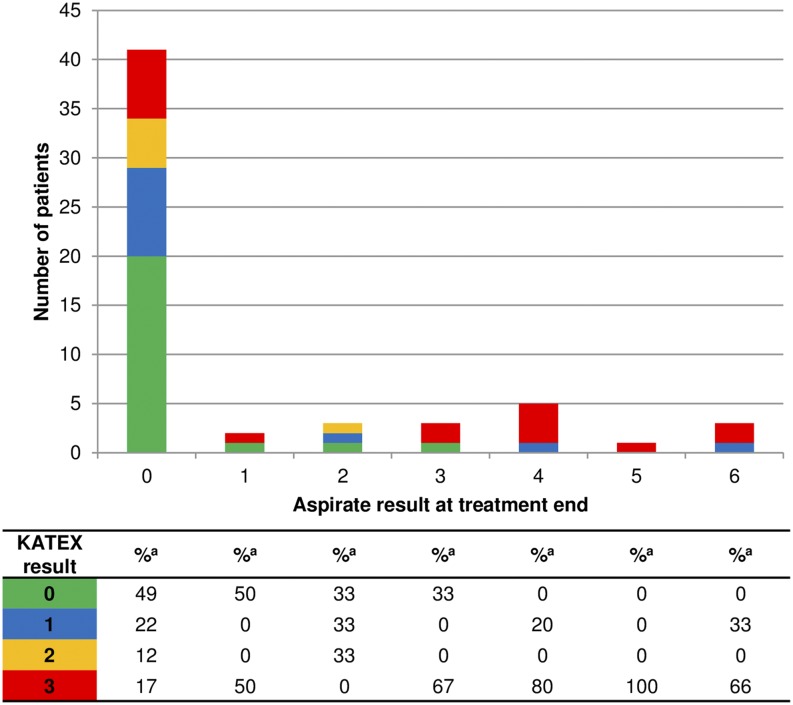
Aspirate and KATEX results at treatment end. Thirteen patients with missing aspirate result at treatment end and 16 patients with missing KATEX result at treatment end excluded (see Supplementary Material 1). ^a^ Percentage of column total. This figure appears in color at www.ajtmh.org.

**Figure 6.**
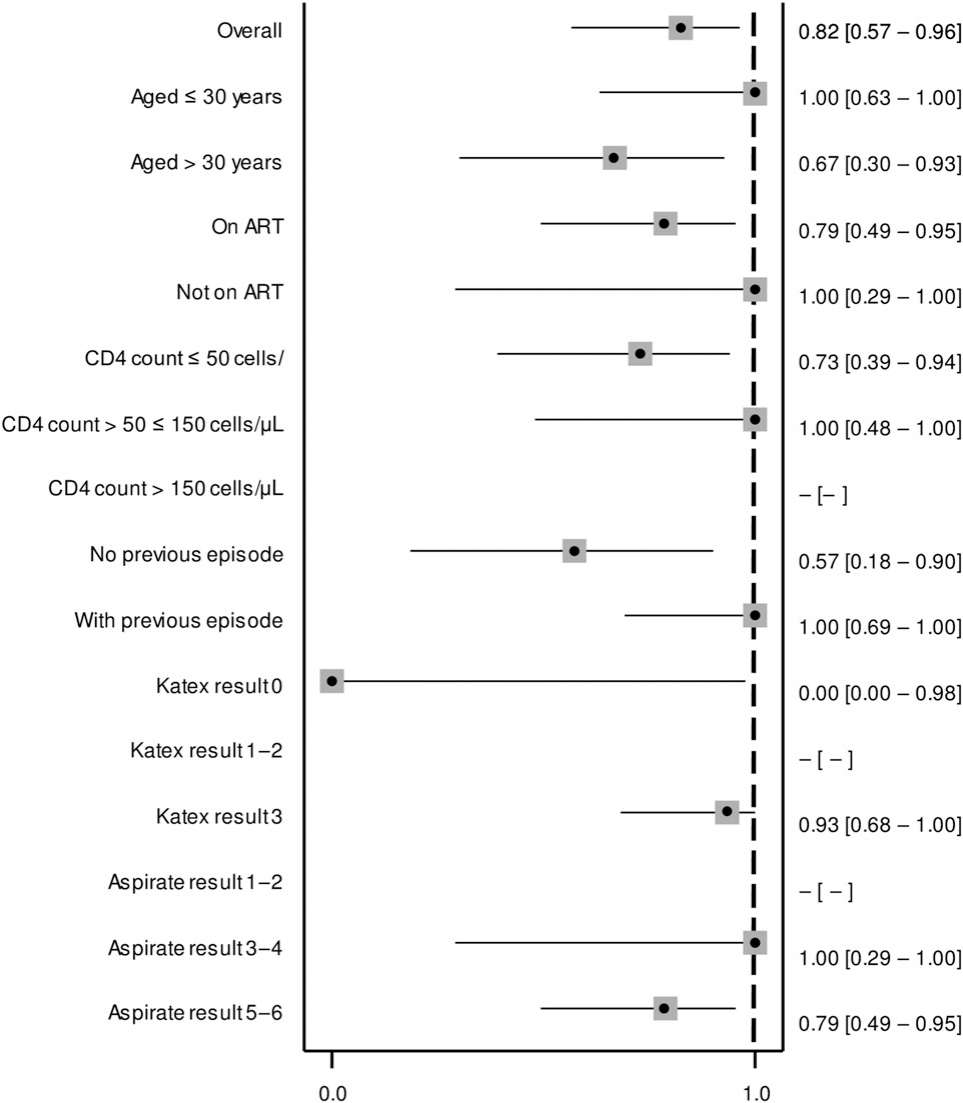
Sensitivity of KATEX at treatment end as test of cure. Thirteen patients with missing aspirate result at the treatment end and 16 patients with missing KATEX result at the treatment end excluded (see Supplementary Material 1). All variables refer to the time of treatment start. 95% confidence intervals in brackets.

**Figure 7.**
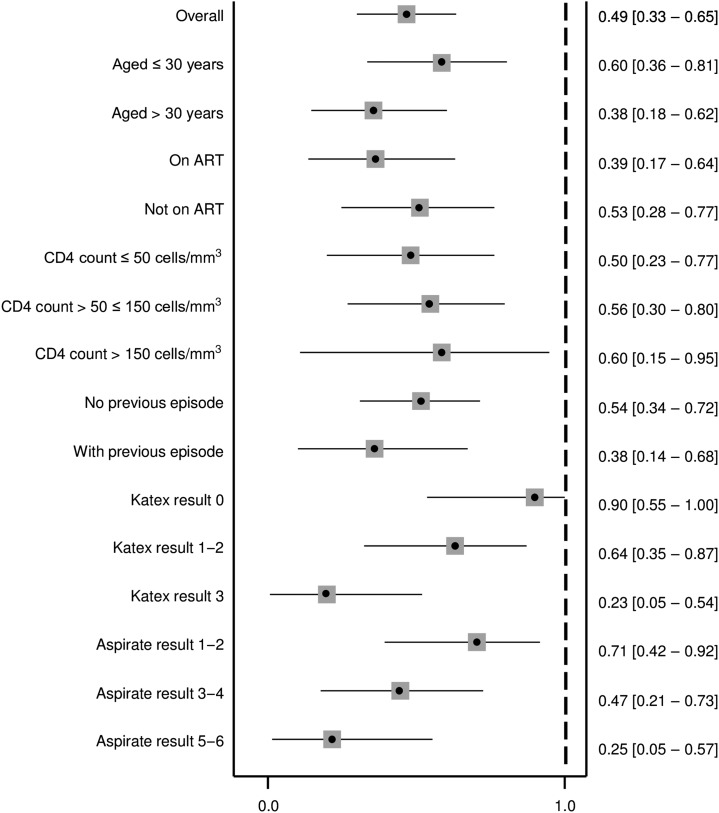
Specificity of KATEX at the treatment end as test of cure. Thirteen patients with missing aspirate result at treatment end and 16 patients with missing KATEX result at treatment end excluded (see Supplementary Material 1). All variables refer to the time of treatment start. 95% confidence intervals in brackets.

**Figure 8.**
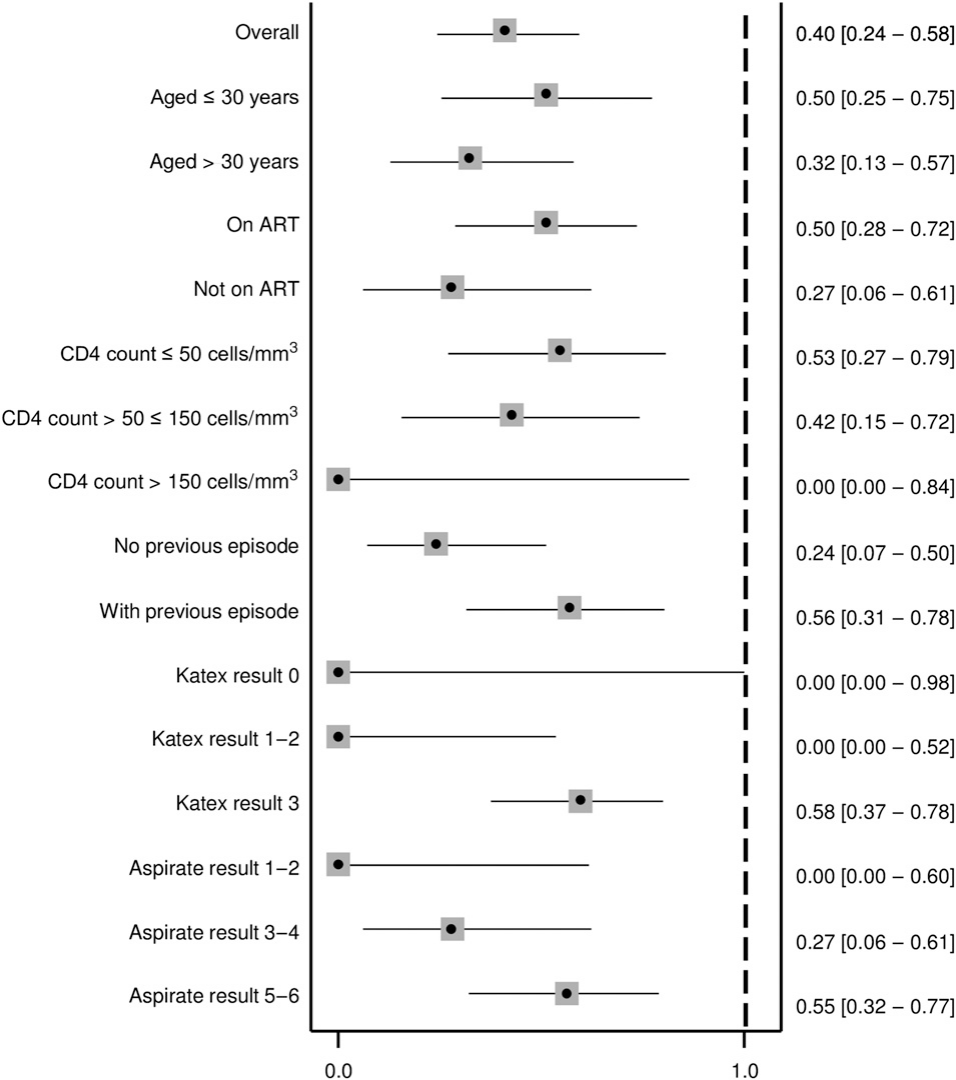
Positive predictive value of KATEX at treatment end as test of cure. Thirteen patients with missing aspirate result at treatment end and 16 patients with missing KATEX result at treatment end excluded (see Supplementary Material 1). All variables refer to the time of treatment start. 95% confidence intervals in brackets.

**Figure 9.**
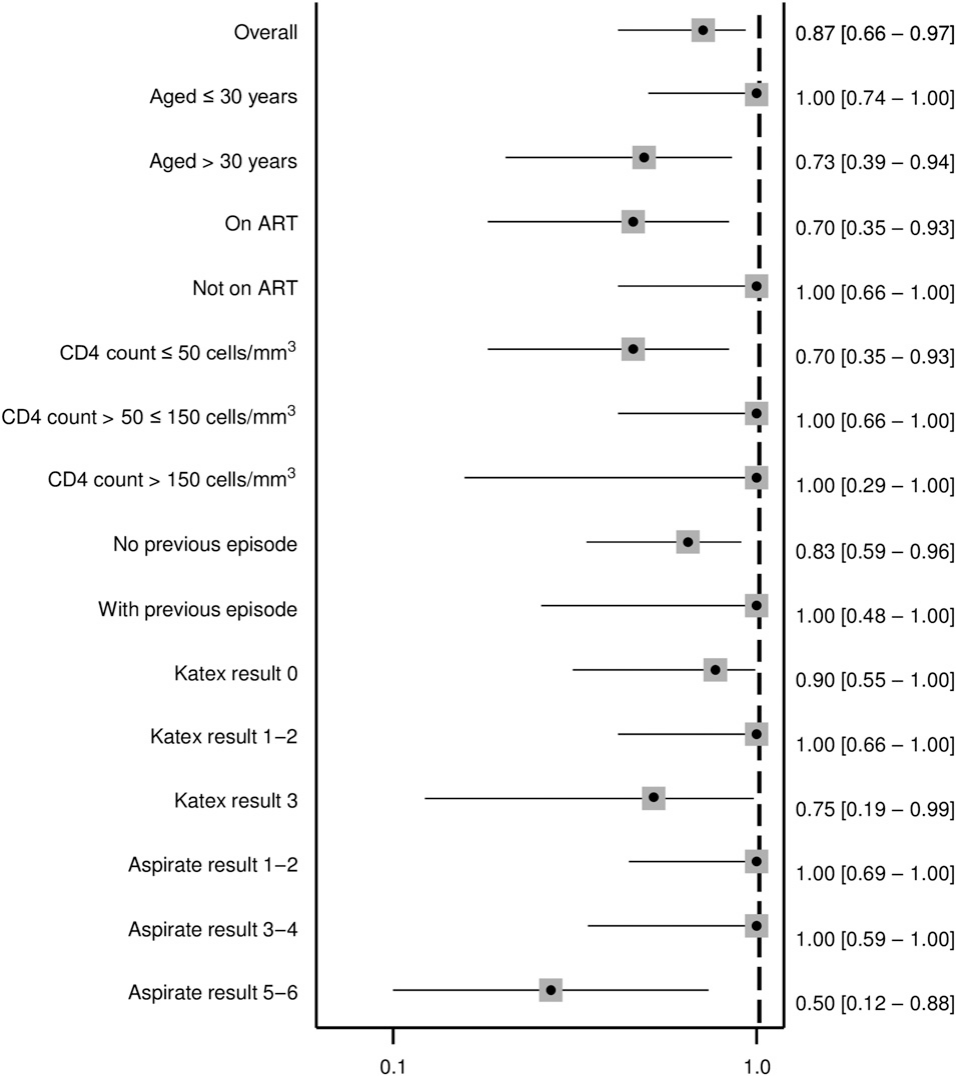
Negative predictive value of KATEX at treatment end as test of cure. Thirteen patients with missing aspirate result at treatment end and 16 patients with missing KATEX result at treatment end excluded (see Supplementary Material 1). All variables refer to the time of treatment start. 95% confidence intervals in brackets.

Similarly as at treatment start, KATEX and aspirate results were also correlated at treatment end. Half of cured patients were KATEX negative, although high (+3) KATEX results dominated in treatment failures with medium and high aspirate loads (Figure 5). The corresponding Spearman’s Rho correlation coefficient was 0.420 (*P* value 0.001). Overall sensitivity of KATEX as TOC was moderate (82%, 95% CI: 5–96), whereas specificity was low (49%, 95% CI: 33–65). The PPV was low (40%, 95% CI: 24–56) and the NPV was moderate (87%, 95% CI: 66–97). No clear pattern emerged among stratified covariates (Figures 6–9).

## DISCUSSION

This was the first study evaluating the potential of KATEX-based antigen detection in urine to diagnose and monitor VL treatment response in HIV-coinfected patients from an *L. donovani*–endemic region.

As hypothesized, we found KATEX to have a moderate sensitivity (84%) for VL diagnosis in HIV-coinfected individuals (Figure 2). Using urine from 97 healthy endemic controls, we also confirmed its high specificity (99%). Similar results have been found in the few existing KATEX evaluations in HIV patients: in one study from Spain, KATEX was positive in all 12 aspirate-confirmed VL patients and negative in 70 of 73 aspirate-negative patients with clinical symptoms of VL (sensitivity 100%, specificity 95%).^22^ In another study from Spain, also among HIV-coinfected patients, sensitivity of KATEX for VL diagnosis was 86% (42/49), with a corresponding NPV of 89% (59/66) and a specificity in asymptomatic controls of 100%.^23^ In Brazil, KATEX was positive at treatment start in three of four aspirate-confirmed HIV-coinfected patients in a small case series^30^ but only positive in one of three cases in another.^31^ One study from *L. donovani*–endemic Sudan evaluating KATEX for VL diagnosis mostly in HIV-negative patients had also two HIV-coinfected patients included. Both of them tested positive for KATEX.^21^ Overall, sensitivity of KATEX appears to be higher in HIV-coinfected patients.^11^ Sensitivity could theoretically be further increased if used in a diagnostic algorithm in combination with other tests such as the rK39 rapid diagnostic test and the direct agglutination test. This aspect warrants further investigation. Combined with its high specificity, KATEX could, thus, have a role to play for VL diagnosis in HIV-coinfected patients at decentralized health-care levels, especially if a test kit in dipstick format could be developed.

As TOC, KATEX performed moderately well in terms of sensitivity (82%) and NPV (87%) in our patients but only poorly for specificity (49%) and PPV (40%) (Figures 6–9). The observed decline in antigen levels during treatment occurred mainly in patients with medium-level parasite loads, whereas patients with very low or very high parasite load at treatment start tended to have relatively stable KATEX levels (Figure 4). Patients with subsequent treatment failure tended to start with high (+3) KATEX levels (Table 2) and continue as such throughout treatment (Figure 4). No comparable studies among HIV-coinfected patients exist on this aspect. In HIV-negative VL patients from Sudan, KATEX was positive at treatment end in all five patients with positive TOC aspirate and negative in 17 of 19 patients with negative TOC aspirate (sensitivity 100% and specificity 85%).^21^ In India, also among HIV-negative patients, eight of 273 patients were found to be KATEX positive at treatment end (specificity 97%).^28^ The most likely explanation for the markedly lower specificity of KATEX as TOC in our study is prolonged antigen secretion due to an impaired immune response. Antigen clearance is dependent on a functioning immune response; hence, immunocompromised patients may continue to shade antigen at VL treatment end despite achieving parasitological cure. Also, tissue invasion by *Leishmania* parasites is more disseminated in immunocompromised patients. Standard spleen or bone marrow aspirates might, thus, be negative, especially when parasite loads have decreased towards the end of treatment, whereas viable parasites might still be present in alternative reservoirs elsewhere in the body.

Most diagnostic studies only report both KATEX and aspirate results as either positive or negative (binary). We assessed both tests in full detail semiquantitatively and found strong positive correlations both at the beginning and at the end of treatment (Figures 1 and 5). A correlation of KATEX positivity and aspirate parasite load at VL diagnosis has also been found in studies from Bangladesh^19^ and Nepal,^20^ although those were not conducted in HIV patients. Among patients with low aspirate (+1) parasite load in our study, 57% and 50% were KATEX negative at VL diagnosis and at TOC, respectively (Figures 1 and 5). This could be due to a failure of KATEX to detect low *Leishmania* antigen levels or due to the absence of *Leishmania* antigen secretion in urine in patients with low tissue parasite loads.

In our study, 33 cured patients and 19 patients with treatment failure remained KATEX positive at TOC (Table 2). There is ambiguous evidence about the use of KATEX during the post-treatment period and for relapse prediction in HIV patients. In one *L. infantum* study from Europe, KATEX remained positive up to 1 year in some HIV-coinfected patients,^22^ suggesting that KATEX was not useful for treatment monitoring and prediction of relapse. However, authors of another HIV coinfection study conclude KATEX results during post-treatment follow-up to be predictive of VL relapse in such patients.^23^ KATEX cannot differentiate between antigen released from lysed (dead) and viable parasites,^29,32^ and it is not known for how long parasite material is secreted in urine after cure.^11^ Unfortunately, no KATEX tests were taken from KATEX-positive patients regularly after their TOC in our study, which would have been necessary to distinguish these conditions. A good correlation between antigen detection by KATEX and culturing parasites from peripheral blood mononuclear cells was found during longitudinal monitoring of asymptomatic HIV patients after VL cure,^23^ supporting the hypothesis that persisting antigenuria results from viable parasites. Patients with HIV infection frequently fail to fully clear the parasite. Such patients can have a negative TOC and can be symptom free, as shown in European HIV patients in whom parasites could be cultured from blood over a period of 10 years after treatment, including during asymptomatic periods.^33^ Since it is recognized that asymptomatic carriers vastly outnumber clinical VL cases,^6,34^ this condition—labeled as “active chronic VL”^33^—raises concerns about infectivity of cured asymptomatic HIV patients.^35^ Future studies should, therefore, compare (combinations of) other novel noninvasive direct parasite detection methods such as peripheral blood microscopy^36^ and quantitative polymerase chain reaction technology^37^ against KATEX, and in particular, cover the post-treatment phase.^13,17,18,38^ Also, more sensitive urine antigen tests for *L. donovani* were recently described but remain yet to be evaluated in HIV-coinfected patients.^15^

This study has some limitations. First, this was an exploratory sub-study embedded in a trial without sufficient sample size to allow meaningful sub-group analyses. Consequently, all findings need to be interpreted with appropriate caution until confirmed in properly powered studies. Second, because the main trial only enrolled VL-confirmed cases, we relied on healthy endemic controls to assess specificity of KATEX for VL diagnosis. Patients with HIV infection and clinically suspected VL symptoms in which the disease had been ruled out would have been preferable. Whereas this could have potentially overestimated specificity in our study, KATEX has been highly specific in virtually all other studies, including among HIV patients, arguing against a major impact on our findings. Third, some of the urine samples were stored for up to 6 years at −20°C before testing, thereby theoretically reducing sensitivity. However, because only a minor reduction of antigen detection after a storage duration of 8 years has been found elsewhere,^20^ it is unlikely that this affected our results substantially. Last, an extension of our study into the post-treatment period and inclusion of other infection markers would have greatly enriched the interpretation of our findings.

In conclusion, our findings suggest that KATEX could contribute to the diagnosis of VL in HIV-coinfected patients, in particular at decentralized health care levels. However, its role for monitoring treatment response seems to be limited, pending further evaluations. Better noninvasive diagnostic options are needed to expand and improve clinical care for VL in resource-limited settings.

## Supplementary Material

Supplemental material
