# Supplementary material for: Antigen Detection in Urine for Noninvasive Diagnosis and Treatment Monitoring of Visceral Leishmaniasis in Human Immunodeficiency Virus Coinfected Patients: An Exploratory Analysis from Ethiopia
Source: Am J Trop Med Hyg. 2018 Aug 6;99(4):957–66. doi: 10.4269/ajtmh.18-0042 (PMC6159592; doi:10.4269/ajtmh.18-0042)
Supplement: Supplementary file 1 [file tpmd180042.SD1.ppt]

## Slide 1
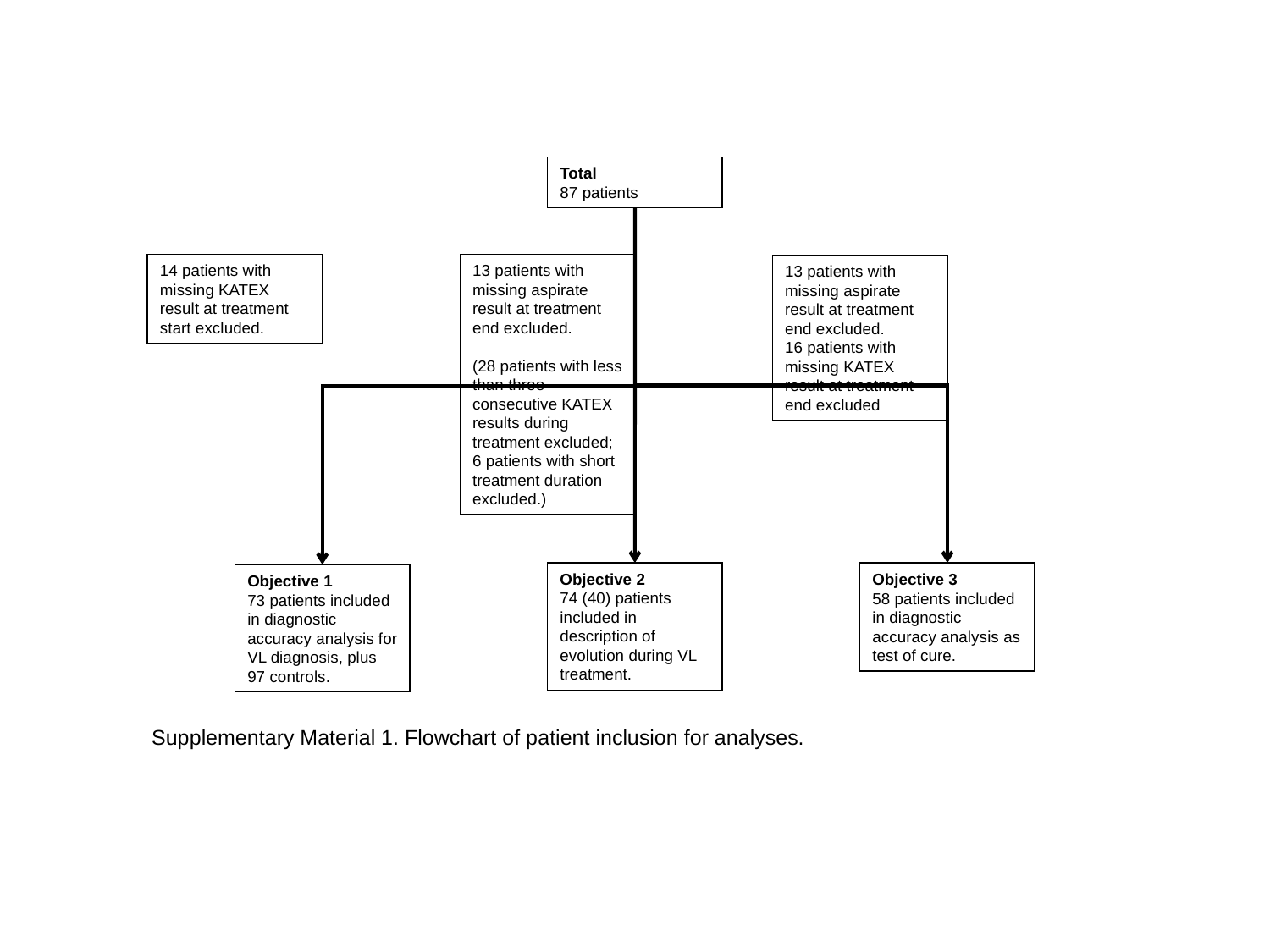

Total
87 patients
14 patients with missing KATEX result at treatment start excluded.
13 patients with missing aspirate result at treatment end excluded.
(28 patients with less than three consecutive KATEX results during treatment excluded;
6 patients with short treatment duration excluded.)
13 patients with missing aspirate result at treatment end excluded.
16 patients with missing KATEX result at treatment end excluded
Objective 2
74 (40) patients included in description of evolution during VL treatment.
Objective 3
58 patients included in diagnostic accuracy analysis as test of cure.
Objective 1
73 patients included in diagnostic accuracy analysis for VL diagnosis, plus 97 controls.
Supplementary Material 1. Flowchart of patient inclusion for analyses.
